# Mode of action of quinoline antimalarial drugs in red blood cells infected by *Plasmodium falciparum* revealed in vivo

**DOI:** 10.1073/pnas.1910123116

**Published:** 2019-10-28

**Authors:** Sergey Kapishnikov, Trine Staalsø, Yang Yang, Jiwoong Lee, Ana J. Pérez-Berná, Eva Pereiro, Yang Yang, Stephan Werner, Peter Guttmann, Leslie Leiserowitz, Jens Als-Nielsen

**Affiliations:** ^a^Niels Bohr Institute, University of Copenhagen, 2100 Copenhagen, Denmark;; ^b^Department of Immunology and Microbiology, Faculty of Health Sciences, University of Copenhagen, 2100 Copenhagen, Denmark;; ^c^Department of Clinical Microbiology, Copenhagen University Hospital, 2100 Copenhagen, Denmark;; ^d^Department of Chemistry, University of Copenhagen, 2100 Copenhagen, Denmark;; ^e^MISTRAL Beamline Experiments Division, ALBA Synchrotron Light Source, 08290 Barcelona, Spain;; ^f^European Synchrotron Radiation Facility, 38000 Grenoble, France;; ^g^Joint Research Group X-Ray Microscopy, Helmholtz-Zentrum Berlin, 12489 Berlin, Germany;; ^h^Department of Materials and Interfaces, Weizmann Institute of Science, 76100 Rehovot, Israel

**Keywords:** malaria, cryo X-ray microscopy, drug tracking, inhibition of crystallization, hemozoin

## Abstract

The most widely used antimalarial drugs belong to the quinoline family. Their mode of action has not been characterized at the molecular level in vivo. We report the in vivo mode of action of a bromo analog of the drug chloroquine in rapidly frozen *Plasmodium falciparum*-infected red blood cells. The *Plasmodium* parasite digests hemoglobin, liberating the heme as a byproduct, toxic to the parasite. It is detoxified by crystallization into inert hemozoin within the parasitic digestive vacuole. By mapping such infected red blood cells with nondestructive X-ray microscopy, we observe that bromoquine caps hemozoin crystals. The measured crystal surface coverage is sufficient to inhibit further hemozoin crystal growth, thereby sabotaging heme detoxification. Moreover, we find that bromoquine accumulates in the digestive vacuole, reaching submillimolar concentration, 1,000-fold more than that of the drug in the culture medium. Such a dramatic increase in bromoquine concentration enhances the drug’s efficiency in depriving heme from docking onto the hemozoin crystal surface. Based on direct observation of bromoquine distribution in the digestive vacuole and at its membrane surface, we deduce that the excess bromoquine forms a complex with the remaining heme deprived from crystallization. This complex is driven toward the digestive vacuole membrane, increasing the chances of membrane puncture and spillage of heme into the interior of the parasite.

## Significance

The most widely used antimalarial drugs belong to the quinoline family. The question of their mode of action has been open for centuries. It has been recently narrowed down to whether these drugs interfere with the process of crystallization of heme in the malaria parasite. To date, all studies of the drug action on heme crystals have been done either on model systems or on dried parasites, which yielded limited data and ambiguity. This study was done in actual parasites in their near-native environment, revealing the mode of action of these drugs in vivo. The approach adopted in this study can be extended to other families of antimalarial drugs, such as artemisinins, provided appropriate derivatives can be synthesized.

Human malaria, a reemerging infectious disease, is caused by several types of protozoan parasites of the genus *Plasmodium*. It has been one of the primary concerns to humanity for centuries and is now extended to more than 40% of the world’s population. Our focus will be on the most virulent of such species, *Plasmodium falciparum*. Increasing geographical spread of the species resistant to current drug treatments is a cause of serious concern (1–3). Characterizing how current antimalarial drugs work at the molecular level is a key for intelligent design of improved antimalarial drugs needed to combat the disease.

As part of its life cycle the *Plasmodium* parasite invades a red blood cell, where it catabolizes hemoglobin to grow and multiply. The hemoglobin, on digestion in the parasitic digestive vacuole, releases iron-containing heme molecules, which are toxic to the parasite. The heme is rendered inert by crystallization into hemozoin. We have recently shown that the parasite stores large quantities of hemoglobin in its digestive vacuole (4). For the parasite to survive, the rate of heme liberation via hemoglobin digestion must not exceed the rate of hemozoin crystallization. Hindering this step would lead to buildup of the toxic heme within the parasite (5, 6).

Among proposed hypotheses, the quinoline-family drugs are believed to damage the parasite by the following steps: 1) via quinoline capping the growing hemozoin crystals, thereby retarding deposition of heme onto the crystal surface (7–10), and 2) complexing with free heme in the lumen of the digestive vacuole (11–14), although this process should be secondary in terms of inhibiting crystal growth (9). The net result in both hypotheses is damage imparted to the parasite by heme released from hemoglobin but unable to crystallize (15, 16).

Thus, to establish the mechanism of antimalarial action by quinoline drugs, it was imperative to determine the crystal structure of hemozoin. A breakthrough was achieved by Pagola et al. (17) nearly 20 y ago characterizing the crystal structure of synthetic hemozoin (see *SI Appendix*, section 1 and Fig. S1), which is composed of heme dimers. Based on this crystal structure it became possible to characterize the faces of hemozoin (*SI Appendix*, Fig. S1*B*) and so devise a model of quinoline drug binding to the {100}, {001}, and {011} faces, as was proposed by Weissbucht and Leiserowitz (7) and Buller et al. (18). Later, Kapishnikov and Leiserowitz, in an article by Biot and coworkers (8), provided an improved model of chloroquine-type binding to the hemozoin {100} face, which involves an acid–base interaction between a quinoline molecule and 2 accessible heme propionic acid groups spanning 2 unit cells on the well-expressed {100} face. However, to date, all growth inhibition studies have been done on synthetic hemozoin.

Various approaches were employed to investigate whether and how such drugs inhibit hemozoin crystallization (9, 18, 19). In vitro studies monitored crystallization of synthetic hemozoin in the presence of drug molecules, whose affinity to the crystal surface and inhibition of crystallization was tested, but in media different from that of the parasitic digestive vacuole (9, 20, 21). A more direct mode of investigation involved localization of the drug ferroquine within the malaria parasite itself (22), albeit in a dried red blood cell.

In order to validate the proposed mechanisms of action of antimalarial quinoline drugs we raised the following questions. What is the concentration of the drug in the digestive vacuole of the parasite? Does the drug indeed bind to the hemozoin crystal faces and persist at these crystal surfaces in vivo? If so, would the crystal surface coverage by the drug be sufficient to inhibit regular heme dimer adsorption onto the crystal faces? Finally, can we detect or envisage binding of the drug to free heme?

**Fig. 1.**
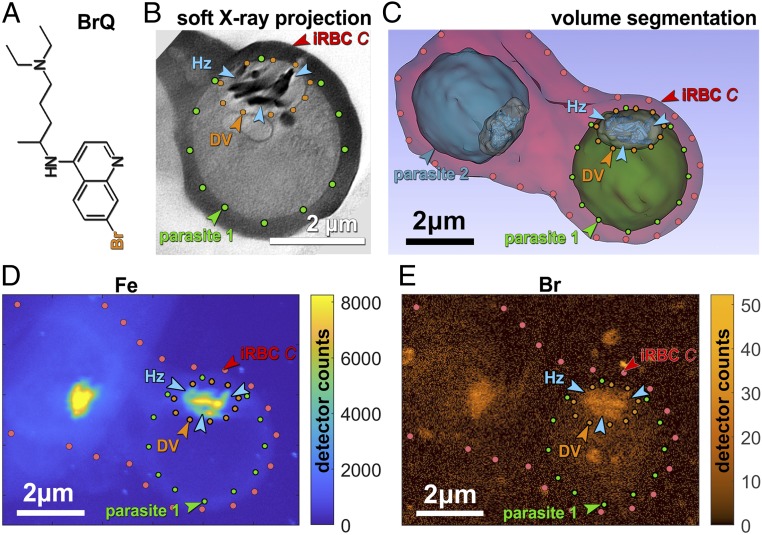
(*A*) Bromoquine molecule, BrQ, where Br takes the place of chlorine of the original drug chloroquine (see *BrQ Synthesis and Methods* for synthesis of BrQ). (*B*) Soft X-ray projection through parasite 1 along the same beam direction as in X-ray fluorescence maps shown in *D* and *E*. For parasite 1 the parasite membrane and the digestive vacuole (DV) are delineated by green and orange dots, respectively. Clusters of hemozoin (Hz) crystals are denoted by blue arrowheads. (*C*) Full segmentation of soft X-ray tomogram of the infected red blood cell (iRBC) labeled “*C*” with different compartments rendered in artificial colors. The 2 parasites that share the same red blood cell are colored green and blue and labeled parasite 1 and parasite 2, respectively. (*D*) X-ray fluorescence map of Fe in iRBC labeled “*C*” containing both parasites. Parasite 1 is delineated in accordance with the delineation in *C*. (*E*) X-ray fluorescence map of Br in the same infected red blood cell with parasite 1 delineated in the same way as in *D.* The similarity between the fluorescence maps of Fe and Br clearly demonstrates the affinity of BrQ molecules for attachment to the Hz crystals. The intensity scale factor *I*_*Fe*_/*I*_*Br*_ ∼ 272 (see main text) with an estimated uncertainty of 15%. The corresponding BrQ coverage is (7 ± 2)%.

With these considerations in mind, we undertook a correlative X-ray microscopy study to establish the mode of action of established drugs like chloroquine via its localization within hydrated malaria parasites in their native, albeit rapidly frozen, environment. We provide evidence that both mechanisms take place and argue that the interaction is sufficient to disrupt heme detoxification. We used bromoquine (BrQ), an analog of chloroquine (cf. Fig. 1*A*), to take advantage of the identifiable X-ray fluorescence signal of the bromine substituent, assuming that BrQ’s molecular biological properties do not differ significantly from that of chloroquine (23, 24). We found that BrQ accumulates at high concentrations in the digestive vacuole, thousandfold exceeding that in the culture medium. Within the digestive vacuole, BrQ caps the hemozoin crystal surface. Such a coverage, defined as the fraction of surface docking sites capped by BrQ and found to range between 4% and 15%, is sufficient to hinder the deposition of oncoming heme molecules. We also observed that BrQ covers hemozoin crystals isolated from the parasites to the same extent as in the digestive vacuole. This suggests a persistent binding of BrQ to the crystal surface. We deduce that BrQ forms a complex with isolated heme, which accumulates at the digestive vacuole membrane, possibly spreading to other parasitic membranes, leading to their disruption.

Based on these in vivo observations, we present in the closing words of this paper a model of the antimalarial mode of action by BrQ and, by extension, that of related quinolines.

## Results

To verify the first hypothesis that quinoline drugs cap hemozoin crystals, we colocalize the iron (Fe) signal of hemozoin crystals with the bromine (Br) signal of BrQ. In order to assess the efficiency of hemozoin growth inhibition by capping with BrQ we measure the BrQ coverage on the surface of hemozoin crystals.

To provide evidence of strong attachment of BrQ to the hemozoin crystal surface we compare BrQ coverage of crystals within the digestive vacuole with those isolated from the parasites. We find a similar coverage in both cases, suggesting strong interaction between BrQ and the hemozoin crystal surface.

To validate that our calculations do not include background Br signal from culture medium, we analyze the Br distribution in BrQ-treated and BrQ-free samples. We find that background Br originating from bovine serum used to culture the parasites does not significantly enter the digestive vacuole.

We identify an increased concentration of BrQ at the membrane of the digestive vacuole. This can be explained by BrQ complexation with free heme (see *Discussion*), thereby validating the second hypothesis that quinoline drugs interact with free heme.

Finally, to verify immediate availability of BrQ at the site of hemozoin nucleation and growth we measure concentration of BrQ within the digestive vacuole but not attached to hemozoin crystals. We find it accumulates in the digestive vacuole at a thousandfold higher concentration than in the culture medium, confirming previously reported estimates (25–27).

### BrQ Caps Hemozoin Crystals in Infected Red Blood Cells.

A red blood cell, which happened to be invaded by 2 parasites (Fig. 1), was treated with BrQ drug 30 h after infection. The cell was nondestructively imaged by soft X-ray cryotomography with the photon energy within the so-called water window range in which oxygen atoms are practically transparent and carbon atoms are heavily absorbing. This generates strong contrast between lipid, cytoplasm, and hemozoin crystals (Fig. 1*B*), enabling direct tomographic mapping of cellular structure within entire parasites without the need for staining.

A sample with infected red blood cells, frozen in 20-μm-thick ice, was mapped by soft X-ray cryotomography. From the tomographic data it was possible to render a complete 3-dimensional (3D) picture of an infected red blood cell, shown in Fig. 1*C* in artificial colors.

The cell was subsequently mapped by X-ray fluorescence cryomicroscopy. This involves raster scanning with a 30-nm-diameter hard X-ray beam, which causes fluorescent X-rays to be emitted uniformly in all directions, with an energy spectrum reflecting the element distribution in the irradiated volume. The scan provides a distribution map for each atomic element, but here we consider only the contributions of Fe and Br as shown in Fig. 1 *D* and *E*. The similarity between the Fe map in Fig. 1*D* and the calculated soft X-ray projection in Fig. 1*B* enables an unambiguous association between Fe and Br distribution and structure of parasite 1, including hemozoin crystals and its digestive vacuole. Clearly, large Br concentrations are located precisely in the same regions as the large Fe concentrations of the hemozoin crystals. Similar overlap has been observed in every BrQ-treated parasite, while no such overlap has been observed in any of the BrQ-free parasites. In total, 7 BrQ-treated and 9 BrQ-free parasites were mapped by X-ray fluorescence cryomicroscopy.

### BrQ Caps One-Tenth of Hemozoin Surface Docking Sites within Parasites.

First, we assume there is no occlusion of the BrQ into hemozoin crystals since no direct evidence was found for the existence of quinine or chloroquine within synthetic hemozoin grown in their presence (21). Hence, BrQ is always located at the hemozoin crystal surface.

Let *N*_*surf*_ be the number of unit cells on the crystal surfaces at which a fraction *C*_*cvrg*_ is covered by BrQ molecules. The number of BrQ molecules at the hemozoin surface is *C*_*cvrg*_·(*N*_*surf*_/2) because 1 BrQ molecule will bind to 2 adjacent surface unit cells on the {100}, {011}, and {001} faces (see Introduction and *SI Appendix*, section 1). We shall, for convenience, assume that the BrQ binds equally well on the different faces since {*h,k,l*} face assignment was not possible due to the limited spatial resolution.

Let *N*_*bulk*_ define the total number of unit cells in the bulk of the hemozoin crystals. The number of Fe atoms constituting the hemozoin crystals is 2·*N*_*bulk*_, because 1 unit cell of hemozoin contains 2 Fe atoms (cf. *SI Appendix*, Fig. S1*A*).

The ratio of the number of BrQ molecules to Fe atoms is then

$$\frac{N_{\text{BrQ}}}{N_{\text{Fe}}}=\frac{C_{cvrg}\left(N_{surf}/2\right)}{2N_{bulk}}=C_{cvrg}\cdot \frac{N_{surf}}{N_{bulk}}\cdot \frac{1}{4}.$$

At this point, we take into account that the measured X-ray fluorescence intensity *I* per atom of Br is 5.4 times higher than that for Fe, as detailed in *SI Appendix*, section 2. Therefore,

$$\frac{N_{\text{BrQ}}}{N_{\text{Fe}}}=\frac{1}{5.4}\cdot \frac{I_{\text{Br}}}{I_{\text{Fe}}}=C_{cvrg}\cdot \frac{N_{surf}}{N_{bulk}}\cdot \frac{1}{4}.$$

From this equation we derive the surface coverage:

$$C_{cvrg}=\frac{4}{5.4}\cdot \frac{I_{\text{Br}}}{I_{\text{Fe}}}\cdot \frac{N_{bulk}}{N_{surf}}.$$

An estimate of the fluorescence signal ratio, $I_{\text{Br}}/I_{\text{Fe}}=40/(8\times 10^{3})=1:200$, can be obtained by inspection of the vertical color scales of the 2 maps (Fig. 1 *D* and *E*). A more precise measure is obtained by summing up the intensities in the Fe map (Fig. 1*D*) within the pixel area of hemozoin crystals and in the same pixel area in the Br map (Fig. 1*E*) with a constant background subtracted. The result is a ratio of 1:272.

What remains to be assessed is the ratio $N_{bulk}/N_{surf}$ for all of the hemozoin crystals in the digestive vacuole. This was done by measuring the volume and surface area of the cluster of hemozoin crystals using soft X-ray tomography datasets, as detailed in *SI Appendix*, section 4.1, resulting in the value $N_{bulk}/N_{surf}=10^{8}/3.9\cdot 10^{6}$ for parasite 1.

We thus estimate the resulting fractional coverage *C*_*cvrg*_ to be

$C_{cvrg}=\frac{4}{5.4}\cdot \frac{I_{\text{Br}}}{I_{\text{Fe}}}\cdot \frac{N_{bulk}}{N_{surf}}=\frac{4}{5.4}\cdot \frac{1}{272}\cdot \frac{10^{8}}{3.9\cdot 10^{6}}=0.07$, that is, 7 ± 3% of available BrQ docking sites (see *SI Appendix*, section 4.1 for the estimate of the error in surface coverage).

For this doubly invaded red blood cell (iRBC labeled *C*, Fig. 1) the BrQ drug was introduced into the parasite culture at a concentration of 150 nM, which is in the range of the therapeutic dose of chloroquine (28).

In another infected red blood cell (iRBC labeled *A* in Fig. 4), the concentration at which BrQ was introduced into the culture was 40 nM, in keeping with previous measurements reported in the literature (22). Based on the same analytical procedure, the coverage onto hemozoin by BrQ was measured to be ∼10 ± 4%. This extent is nearly the same as that for iRBC *C*, even though the drug was introduced at an almost 4 times lower concentration.

### Isolated Hemozoin Crystals outside Red Blood Cells Are Capped with Bromoquine.

**Fig. 2.**
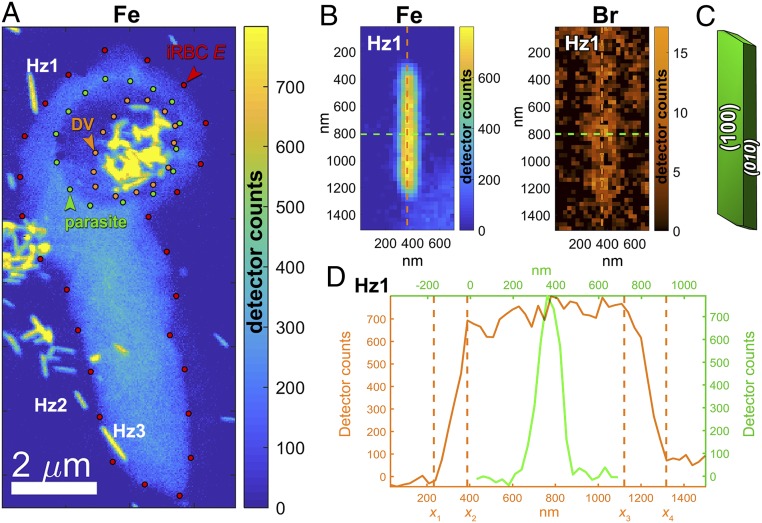
(*A*) Fe X-ray fluorescence map showing a cluster of Hz crystals grown in an infected red blood cell (iRBC) labeled *E* prior to introduction of the BrQ drug, as well as several free-floating Hz crystals labeled Hz1, Hz2, and so on outside the red blood cell. There is a weak (light bluish) Fe signal in the red blood cell originating from hemoglobin still not digested by the parasite. (*B*) Magnification of the Hz1 map showing Fe signal (*Left*) and bromine (Br) signal (*Right*). (*C*) The theoretical growth form of Hz crystals (18) emphasizing that the {100} and {010} are the prevailing faces. (*D*) Section through the Fe map shown in *B*, along the crystal needle axis (in orange) and transverse to it (in green).

The Fe X-ray fluorescence map shown in Fig. 2 reveals several isolated hemozoin crystals outside infected red blood cells. Regarding the origin of these ex vivo crystals, we present 2 scenarios. The crystals may have originated from rupture of previous-generation parasites in the process of release of their daughter parasites at the end of the parasitic asexual red blood cell cycle (29). It is also possible that these crystals were released from those few parasites that were destroyed by BrQ. These ex vivo crystals are marked Hz1, Hz2, and so on in Fig. 2*A*. A close-up view of Hz1 is shown both as Fe and Br maps in Fig. 2*B*, providing prima facie evidence that the BrQ molecule has a distinct affinity to be absorbed onto the surface of hemozoin crystals.

Below we present a determination of BrQ coverage on crystal Hz1, making once again use of the equation

$$C_{cvrg}=\frac{4}{5.4}\cdot \frac{I_{\text{Br}}}{I_{\text{Fe}}}\cdot \frac{N_{bulk}}{N_{surf}}.$$

We have also analyzed 7 other samples of isolated hemozoin crystals (shown in *SI Appendix*, Fig. S3) and incorporate all 8 measurements (*SI Appendix*, Table S1 in *SI Appendix*, section 5) for estimating the average coverage. This estimate is along the same lines as for the analysis within an infected red blood cell (discussed above). However, all isolated crystals were outside the focal plane for soft X-ray tomography measurements, so the determination of surface area *S*_*Hz*_ of each crystal was carried out solely by an analysis of its X-ray fluorescence maps, as detailed in *SI Appendix*, section 4.2. Briefly, the number of unit cells in the bulk of the crystal, $N_{bulk}$, was derived from the number of Fe atoms comprising the crystal measured by X-ray fluorescence with the knowledge that the unit cell of hemozoin consists of 2 Fe atoms. The number of surface unit cells $N_{surf}$ was derived from the crystal surface divided by the weighted average area of surface unit cells. For crystal Hz1 the ratio $N_{bulk}/N_{surf}$ was determined to be equal $1.2\cdot 10^{6}/1.5\cdot 10^{5}$. See *SI Appendix*, section 4.2 for the detailed calculation.

The average X-ray fluorescence intensities of Br and Fe across Hz1 are, *I*_Br_ = 15 and *I*_Fe_ = 700, respectively (Fig. 2*B*). The coverage of BrQ on crystal Hz1 is then

$$C_{cvrg}=\frac{4}{5.4}\cdot \frac{I_{\text{Br}}}{I_{\text{Fe}}}\cdot \frac{N_{bulk}}{N_{surf}}=\frac{4}{5.4}\cdot \frac{15}{700}\cdot \frac{1.2\cdot 10^{6}}{1.5\cdot 10^{5}}\approx 0.12.$$

The same analysis for all 8 isolated crystals yielded an average BrQ coverage of around 0.070 ± 0.035, that is, 7 ± 3.5%. Further experiments with a higher spatial resolution are required to determine the different affinities of BrQ to the {100} and the {010} faces of hemozoin (8).

### Bromine from Bovine Serum Does Not Cap Hemozoin Crystals.

We have identified 2 sources of Br atoms in the examined samples. One is the BrQ drug introduced at the ∼100 nM level. The second source is background Br originating from bovine serum used for culturing the malaria parasites, reportedly present in the form of 2-octyl γ-bromoacetoacetate (30, 31). Measured concentrations of the background Br range up to 4 μM. As shown below, we have been able to effectively separate the total Br signal arising from BrQ and the serum and establish unambiguously that only BrQ decorates hemozoin crystals and the digestive vacuole membrane.

Inspection of the Fe map and the Br map in Fig. 1 shows clearly that Br tends to accumulate on hemozoin crystals, both for parasites 1 and 2. There is also some lower Br signal in the parasites but outside their hemozoin clusters. This signal may arise from the drug BrQ as well as from Br in the serum. In order to distinguish between these 2 sources of Br, we analyze Br distribution in BrQ-treated and the BrQ-free parasites.

**Fig. 3.**
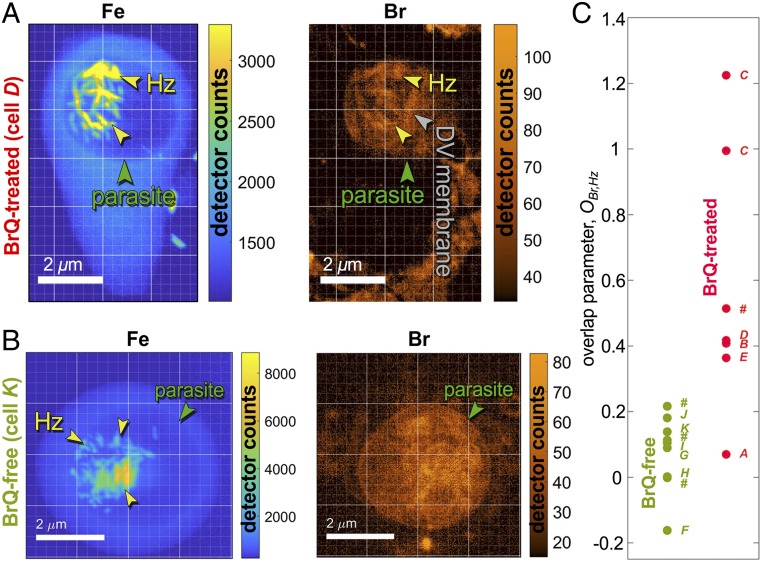
Overlap between Fe and Br distribution in BrQ-treated samples and BrQ-free samples. (*A*) Fe and Br X-ray fluorescence maps of an infected red blood cell (iRBC *D*) treated by BrQ. DV denotes the digestive vacuole and Hz the hemozoin crystals. (*B*) Fe and Br signals in a BrQ-free infected red blood cell (iRBC *K*).(*C*) Overlap parameter, *O*_*Br,Hz*_, between hemozoin position and Br fluorescence signal in 7 BrQ-treated and 9 BrQ-free samples. Each dot represents a measurement within an individual parasite carrying the name of its host iRBC. iRBCs named *A* through *K* are shown in *SI Appendix*, Fig. S6; others are labeled with a hash mark. iRBCs *C* and *A* are shown in Figs. 1 and 2, respectively. The overlap parameter = 0 if there is no overlap between Br and hemozoin and >0 if there is such an overlap.

We analyzed 7 BrQ-treated and 9 BrQ-free parasites. In BrQ-treated parasites, we identify distinct areas of Br overlaying hemozoin (Figs. 1 and 3*A*). In BrQ-free parasites there is no such overlay (Fig. 3*B*). However, the difference in overlay is not always as clear to the naked eye as shown in other examples given in *SI Appendix*, Fig. S6. We have taken up this challenge by quantifying the extent of overlap of Br with hemozoin in BrQ-treated and BrQ-free parasites. This extent is given in form of an overlap parameter *O*_*Br,Hz*_. When there is no overlap *O*_*Br,Hz*_ = 0. In case there is an overlap *O*_*Br,Hz*_ > 0. The overlap parameter for several BrQ-treated and BrQ-free parasites is plotted in Fig. 3*C*. The exact definition and calculation of the overlap parameter are presented in *SI Appendix*, section 9.

For 9 BrQ-free cells *O*_*Br,Hz*_ varied between −0.2 and 0.2, the olive-colored dots in Fig. 3*C*. In the cell shown in Fig. 1, where the overlap is obvious to the eye, the overlap parameter came out to be *O*_*Br,Hz*_ > 1. This is recorded in Fig. 3*C* by 2 red dots marked “*C*” belonging to the 2 parasites residing in this doubly invaded cell. Therefore, the figure shows quantitatively that, although not always as visible as in Fig. 1, there is indeed overlap between Br signal and Hz crystals in BrQ-treated cells, while no such overlap occurs in the BrQ-free samples.

The average overlap parameter in the BrQ-treated cells is 0.57 ± 0.40. In the BrQ-free cells it is 0.09 ± 0.12.

### BrQ Accumulates at the Membrane of the Digestive Vacuole.

**Fig. 4.**
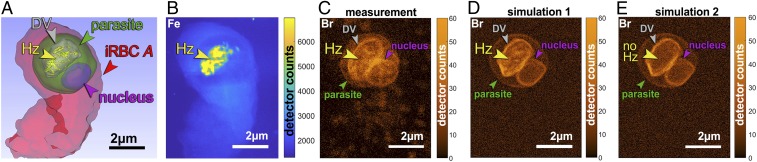
Surface rendering, measured and simulated X-ray fluorescence maps of a BrQ-treated infected red blood cell (iRBC) labeled *A*. (*A*) Surface rendering of a soft X-ray tomography segmentation. (*B*) Measured Fe X-ray fluorescence map. (*C*) Measured Br X-ray fluorescence map. (*D*) Simulated Br X-ray fluorescence map. Br atoms were evenly distributed over the surface of the digestive vacuole (DV) membrane, the parasite nucleus, and the parasite membrane with the density of 5 × 10^3^ atoms per square micrometer, and on the surface of hemozoin (Hz) crystals with a density corresponding to 10% BrQ surface coverage. (*E*) Same simulation as *D* but without Br at the surface of Hz crystals.

In most of the parasites examined, an elevated Br X-ray fluorescence signal appears to decorate the parasitic membranes, including the parasitic nucleus. Br also appears to decorate the digestive vacuole but only in BrQ-treated parasites. In order to examine whether it is the membranes that are decorated by Br, a Br distribution image was simulated by virtually placing Br atoms along the 3D positions of the membranes of the digestive vacuole, the nucleus, and the parasite as shown in Fig. 4. In order to accomplish this task, we have made a crude estimate of Br membrane coverage by summing the Br atoms along the periphery of the digestive vacuole membrane (seen in Fig. 4 *A* and *C*) and dividing this value by measured area of the selected membrane at the corresponding coordinates. This resulted in a Br coverage of 5 × 10^3^ atoms per square micrometer of the membrane.

The 3D coordinates of the parasite membranes were obtained from soft X-ray tomographic reconstruction of the same cell whose lipid membranes are well-resolved. The striking resemblance of the simulated (Fig. 4*D*) and the measured Br distribution (Fig. 4*C*) indicates that the signal comes from a relatively uniform distribution of the Br atoms along the membranes.

BrQ capping of hemozoin crystals can also be confirmed by comparing the measured Br map (Fig. 4*C*) and 2 simulated Br distributions: with and without BrQ capping hemozoin crystals (Fig. 4 *D* and *E*, respectively). Other parasites with similar trend in Br distribution are shown in Fig. 3 and *SI Appendix*, Fig. S6.

A closer inspection of parasite membranes seen in Br maps in Fig. 3 and *SI Appendix*, Fig. S6 reveals that the digestive vacuole membrane is decorated by Br only in BrQ-treated parasites with the digestive vacuole membrane well-delineated in 4 parasites occupying iRBCs *A*, *B*, *D*, and *E*. We therefore conclude that the Br signal at hemozoin crystals and the digestive vacuole membrane indeed originates from BrQ and not from Br found in bovine serum.

Similar observations were made by Dubar et al. (22), who reported the presence of the quinoline drug, ferroquine, close to the digestive vacuole membrane of a *Plasmodium*-infected red blood cell imaged by transmission electron microscopy, and by Woodland et al. (32), who used visible light fluorescence microscopy to locate covalently labeled chloroquine at the digestive vacuole membrane and other parasitic membranes.

### BrQ Reaches High Concentrations in the Digestive Vacuole.

We measured concentration of Br in the lumen of the parasitic digestive vacuole in BrQ-treated parasites by counting Br atoms in 4 infected red blood cells (iRBCs *A*, *B*, *D*, and *E* shown in *SI Appendix*, Fig. S6) in regions within their digestive vacuoles outside hemozoin crystals. The number of Br atoms was divided by the corresponding volume within each of the digestive vacuoles yielding Br concentrations. This concentration reaches a magnitude similar to what has been reported for chloroquine (25–27). We found that BrQ accumulated in the digestive vacuoles, reaching concentrations of 150 ± 50 μM, even though BrQ was introduced into the culture medium at the concentrations as low as 40 nM and 150 nM. The concentration of BrQ in the digestive vacuole was measured in a more direct manner than previously reported for chloroquine, albeit in fewer cells.

## Discussion

We investigate the mode of quinoline-family drug action within *Plasmodium*-infected red blood cells. We have conducted a quantitative measurement of in vivo distribution of the antimalarial drug BrQ within fully hydrated, rapidly frozen, *P. falciparum*-infected red blood cells in the trophozoite stage. These cells are not subject to any artifact deriving from dehydration, staining, chemical fixation, or sectioning and thus provide the closest scenario to an unperturbed biological picture of the drug intervention in the infected red blood cells. Native distribution of chemical elements was measured in the vitrified infected cells at specific time points of parasitic development and drug action.

An elevated presence of bromine atoms was detected within the *Plasmodium* parasitized cells. No bromine signal above background level (set by the concentration of Br atoms in the bovine serum) was detected either within the cytosol compartment of the red blood cell or at its membrane. This observation suggests that once the quinoline molecule reaches the *Plasmodium* parasite, having penetrated the red blood cell, it stays therein. Importantly, BrQ accumulates in the digestive vacuole, reaching concentrations as high as 150 μM, that is, thousandfold higher than the concentration introduced into the culture medium. It is by virtue of the fact that the digestive vacuole is an acidic organelle with pH in the range of 4.5 to 4.9 (33) that BrQ, being a weak base, accumulates therein at high concentrations, as has been earlier suggested for chloroquine (18, 27).

Here we have established that BrQ accumulates at the hemozoin crystal surface and at the digestive vacuole membrane, and possibly at other membranes of the parasite. The crystal surface coverage, measured as a fraction of its surface binding sites blocked by the drug, is between 4% and 15% analyzed for 2 parasites and 8 isolated crystals. We note the presence of BrQ in the lumen of the digestive vacuoles in regions outside the hemozoin crystals at submillimolar concentrations (discussed above), enough to cover an additional 7% of the hemozoin crystals. This result suggests that the BrQ coverage on hemozoin had reached a maximum. BrQ was also found capping large hemozoin crystals floating in the culture medium outside infected red blood cells in which they have been formed. The measured BrQ coverage at these hemozoin surfaces is also within the range of 4% and 15%. This observation confirms strong affinity of BrQ to the hemozoin crystal surface.

Based on computational analysis (8, 18), we conclude that the preferred affinity of BrQ toward the hemozoin crystal surface is likely due to a stereospecific match and attraction between the quinoline molecule and the large {100} side faces and fast-growing {011} and {001} end faces. An essentially homogeneous 10% surface coverage by BrQ should certainly inhibit crystal growth since any oncoming heme dimer will be within 2 unit cell distances of an adsorbed BrQ molecule, and so susceptible to forces unfavorable for docking at a regular surface lattice site on the hemozoin crystal. Such an inhibition would lead to accumulation of the chemically aggressive heme liberated from hemoglobin, resulting in poisoning of the parasite.

We now address the question of the ability of BrQ to smother the hemozoin crystalline surface in the early stages of parasitic development, say a couple of hours after hemozoin crystals begin to form. In a 2-fL digestive vacuole of a young trophozoite (*SI Appendix*, Table S1; electron microscopy data in ref. 34), assuming that half the digestive vacuole volume would be freely occupied by BrQ at 150 μM concentration, the amount of BrQ would be ∼90,000 molecules. This amount would be sufficient to block 11% of hemozoin crystal surface developed at this stage (*SI Appendix*, section 11), implying, all in all, that BrQ would be efficient all the way from the onset of parasitic production of hemozoin crystals.

We now address the observation of an elevated BrQ presence at the membrane of the digestive vacuole. This membrane is clearly visible in soft X-ray tomographic images. In the BrQ-treated parasite the membrane is highlighted in X-ray fluorescence maps of Br, indicating the presence of BrQ molecules. The presence of background Br in parasites precludes analysis of BrQ molecule distribution in other parasitic membranes. Nonetheless, in a recent study by Woodland et al. (32), chloroquine molecules covalently labeled for light fluorescence microscopy were detected also in other parasitic membranes, but not on red blood cell membranes, which is in accordance with our observations. We note that in their work little signal from the labeled molecule over hemozoin-rich area was detected, which is possibly due to signal absorption by the crystals. It is debatable whether the water-soluble drug would, by itself, tend to accumulate at the lipid membranes. However, the drug molecule has high affinity to complex with free heme in the aqueous medium of the digestive vacuole with the acidic pH. Indeed, the possibility of quinoline drug complexation with free heme has been proposed in the literature (7–16). The affinity to water of this complex is presumably higher than that of free heme but lower than that of BrQ alone. We therefore rationalize that this complex would be driven toward and accumulate at the lipid membrane of the digestive vacuole. Supporting this hypothesis is a report of chloroquine–hemin complex association with lipid membranes observed in vitro (35–37). A prolonged exposure of the digestive vacuole membrane to an increased presence of BrQ–heme complex might lead to intercalation of the heme or the complex with the membrane, leading to its local puncture and spread of the toxic heme into the interior of the parasite (36, 37).

We found that BrQ reaches the same concentrations in the digestive vacuole of chloroquine-resistant strain FCR3 as in the BrQ-sensitive 3D7 strain and displays similar distribution in both strains (*SI Appendix*, Fig. S6). We interpret this observation as either lack of resistance to BrQ by the FCR3 strain or a resistance mechanism other than the previously suggested reduction of BrQ concentration in the digestive vacuole (1). Further investigation needs to be conducted to clarify this point.

## Conclusion

Using a correlative X-ray microscopy approach, we have identified that in vivo BrQ—the bromo analog of chloroquine—covers a substantial part of available docking sites at the surface of hemozoin crystals formed in the digestive vacuole of the *Plasmodium* parasites. Quantification of quinoline-type molecule in vivo coverage of hemozoin crystals in fully hydrated cells was achieved. This coverage was as high as 10 ± 4%, enough to prevent oncoming heme docking onto the crystals. The drug was found in abundance in the digestive vacuole. We have provided evidence, albeit indirect, that the drug complexes with free heme, given that the drug binds to the hemozoin surface. This complex accumulates at the membrane of the digestive vacuole, as observed by bromine X-ray fluorescence signal, and possibly spreads to other membranes. In other words, both hypotheses mentioned in the Introduction take place in vivo. This model can be generalized to quinoline drugs, such as quinine, which can stereospecifically bind to the {100}, {011}, and {001} faces of hemozoin.

Last, but not least, the approach described here would be applicable to test whether other antimalarial drugs, such as the widely used artemisinin as an in vivo adduct with heme (38), will bind to hemozoin crystals (21, 39), provided an appropriate atom detectable by X-ray fluorescence can be attached to the drug.

## BrQ Synthesis and Methods

Synthesis of BrQ and the NMR spectrum of the synthesized product are given in *SI Appendix*, section 12.

Malaria parasite culture and strain verification are described in *SI Appendix*, section 13. Tight synchronization resulting in 30- to 32-h-postinvasion parasites is detailed in *SI Appendix*, section 14.

IC_50_ (concentration that inhibits parasite growth by 50%) values of chloroquine and BrQ for chloroquine-sensitive (3D7) and chloroquine-resistant (FCR3) strains and the measurement procedure are given in *SI Appendix*, section 15.

Preparation of vitrified samples for cryo X-ray microscopy is described in *SI Appendix*, section 16. Cryo X-ray fluorescence and soft X-ray cryotomography instruments are described in *SI Appendix*, section 17. Software used in data analysis and presentation is described in *SI Appendix*, section 18.

## Supplementary Material

Supplementary File
