# Supplementary material for: Mode of action of quinoline antimalarial drugs in red blood cells infected by Plasmodium falciparum revealed in vivo
Source: Proc Natl Acad Sci U S A. 2019 Oct 28;116(46):22946–52. doi: 10.1073/pnas.1910123116 (PMC6859308; doi:10.1073/pnas.1910123116)
Supplement: Supplementary File [file pnas.1910123116.sapp.pdf]

Mode of action of quinoline antimalarial drugs in red blood cells infected by *Plasmodium falciparum* revealed *in-vivo*.

Sergey Kapishnikov[a,1], Trine Staalsø[b,c], Yang Yang[d], Jiwoong Lee[d], Ana J Pérez-Berná[e], Eva Pereiro[e], Yang Yang[f], Stephan Werner[g], Peter Guttmann[g], Leslie Leiserowitz[h], Jens Als-Nielsen[a]

[a] Niels Bohr Institute, University of Copenhagen, Universitetsparken 5, 2100 Copenhagen, Denmark
[b] Department of Immunology and Microbiology, Faculty of Health Sciences, University of Copenhagen, 2100 Copenhagen, Denmark
[c] Department of Clinical Microbiology, Copenhagen University Hospital, 2100 Copenhagen, Denmark
[d] Department of Chemistry, University of Copenhagen, Universitetsparken 5, 2100 Copenhagen, Denmark
[e] ALBA Synchrotron Light Source, MISTRAL Beamline–Experiments Division, 08290, Cerdanyola del Valles, Barcelona, Spain
[f] European Synchrotron Radiation Facility (ESRF), 71 avenue des Martyrs, 38000, Grenoble, France
[g] Joint Research Group X-ray Microscopy, Helmholtz-Zentrum Berlin, Albert-Einstein-Str. 15, 12489, Berlin, Germany
[h] Department of Materials and Interfaces, Weizmann Institute of Science, Rehovot, 76100, Israel

[1] Corresponding author. Email for correspondence: sergey.kapishnikov@weizmann.ac.il

Supporting Information

§1. *Hemozoin crystal theoretical growth habit and packing arrangement.*
The dimensions of the triclinic unit cell of hemozoin measured at the temperature of 80K are a = 1.208 nm, b = 1.462 nm, c = 0.799 nm, α = 90.76°, β = 97.09°, γ = 97.06°, V_{uc} =1.390 nm^3, as reported by Straasø *et al.*(1)

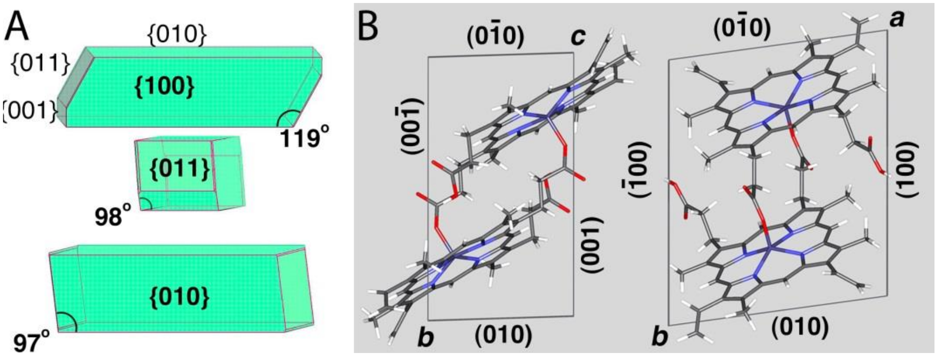

Fig. S1. (A) Theoretical growth habit of hemozoin, displaying the dominant faces (B) Packing arrangement of synthetic hemozoin. Reprinted from ref. (2).

§2. *Relative X-ray fluorescence signal per atom of bromine and iron.*

The measured X-ray fluorescence intensity *per atom* of Br is higher than that for Fe for two reasons. The X-ray fluorescence signal depends on the probability of absorption (3) of incoming X-rays by an atom which increases with the atomic number Z by roughly Z^4, *in casu* $(35/26)^4$=3.28, although a more accurate value of 2.98 can be derived from Tables (4). The other reason is that the fluorescence yield subsequent to an absorption process also rises with the atomic number. It is 0.63 for Br, but 0.35 for Fe (ref. (5)), so altogether the fluorescent intensity per Br atom is higher than per Fe atom by a factor of $2.98 \cdot 0.63/0.35 = 5.4$

§3. *Calculation of the average area of a surface unit cell of hemozoin.*

Hemozoin unit cell areas, in nm^2, with the corresponding Miller planes indicated in the subscript are: A_{100}=1.169, A_{010}=0.959, A_{011}=1.974, A_{001}=1.754.

The relative fraction f that each symmetric pair of crystalline faces occupies on the surface of hemozoin is obtained from its theoretical growth form (see Fig. S1 and ref. (6)) as follows: $f_{\{100\}}$=0.44, $f_{\{010\}}$=0.48, $f_{\{011\}}$=0.06, $f_{\{001\}}$=0.02. The {100} and {010} faces correspond to a 0.92 fraction (i.e. 92%) of the hemozoin crystal surface.

Hence, the average area of a surface unit cell of hemozoin is calculated as $\bar{A}_{\mathrm{Hz}} = 1.169 \cdot 0.44 + 0.959 \cdot 0.48 + 1.974 \cdot 0.06 + 1.754 \cdot 0.02 = 1.3\ \mathrm{nm}^2$

§4.1 *Number of crystalline unit cells on the surface and in the bulk of hemozoin crystals in malaria parasites*

Segmentation of the soft X-ray tomography data has provided a numerical set of cubic voxels belonging to the hemozoin crystals, whose total volume V_{bulk}=1.44·10^8 nm^3 was determined therefrom. Given the volume of the unit cell of hemozoin v_{uc}=1.390 nm^3 (cf. SI Appendix §1), the number of unit cells in the bulk of all hemozoin crystals is $N_{bulk} = \frac{V_{bulk}}{v_{uc}} = \frac{1.44 \cdot 10^8}{1.39} \approx 10^8$

In order to calculate the total surface area of hemozoin crystals, the cubic voxel set was divided into one-voxel-thick slices. One such slice is shown in Fig. 1B. The peripheral length of hemozoin in this two-dimensional pixel set of a slice was measured. Adding up the length of all the peripheries in all the slices through the hemozoin crystals yielded a total surface area of A_{surf} = 4.3·10^6 nm^2.

The weighted average area of surface unit cells on all the principal faces of hemozoin calculated from hemozoin cell dimensions (given in SI Appendix ref. (1) and in SI Appendix §1) is a_{uc}=1.13 nm^2, as detailed in SI Appendix §3. Thus, the total number of unit cells on the hemozoin surface is

$$N_{surf} \approx \frac{A_{surf}}{a_{uc}} = \frac{4.3 \cdot 10^6}{1.13} = 3.9 \cdot 10^6$$

Possible uncertainties in estimates of surface area, volume calculations and surface coverage by BrQ in parasites.

The precision of the surface coverage estimate depends on the precision of measured hemozoin surface area and bromine signal. There are two primary factors that affect

precision of surface area measurement: (1) missing wedge distortion and (2) limited resolution. The third component is (3) the precision of Br signal measurement.

1) Missing wedge elongation

First is the missing wedge distortion in soft X-ray tomographic data due to limited angular span over which tomographic projections were collected. Most of the tomograms were collected over approximately 120° span. The effect on volume estimation was measured from a set of projections of a phantom with three compartments of known volumes, generated over entire 180° and the limited 120° span. The set of projections was reconstructed into a three-dimensional map of the phantom by weighted back projection. A surface rendering of the reconstructed volume for the two datasets is shown in Fig. S2. The increase in volume of each of the three compartments due to the missing wedge is estimated at maximum 3.1%.

In general the surface area relates to volume as $A = fV^{\frac{2}{3}}$, where f depends on the shape of the object. By differentiation $dA = fV^{-\frac{1}{3}}dV$, i.e. $dA/A = \frac{2}{3}dV/V$. Hence the relative uncertainty in surface area is 2/3 of the relative uncertainty of volume. In our case, this is ~**2%**.

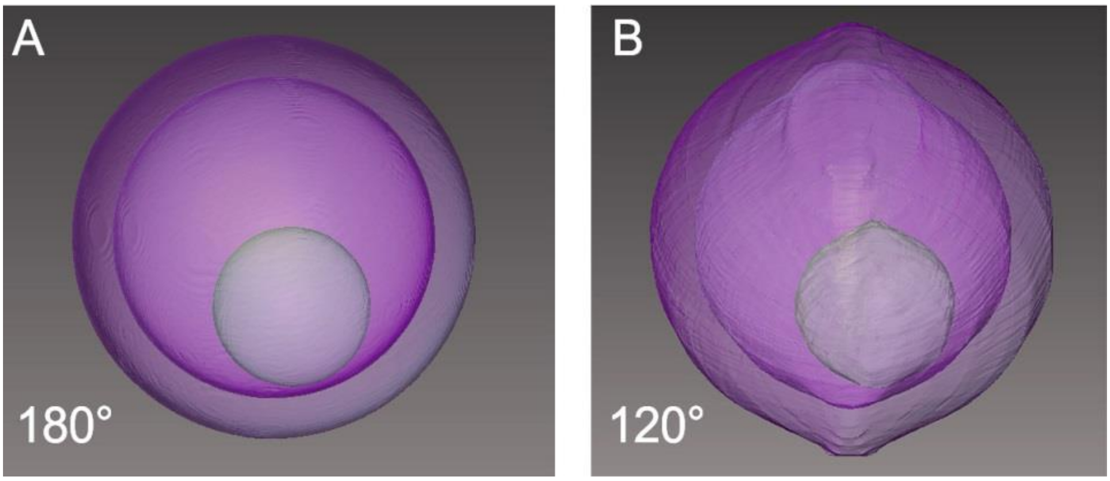

Fig. S2. Surface rendering of weighted back projection reconstruction of phantom projections collected over the angular span of (*A*) 180°, where there is no volume distortion due to the missing wedge and (*B*) 120°, with visible distortion due to the missing wedge.

2) Limited resolution

Second factor is the limited spatial resolution of soft X-ray tomography collected at ALBA and BESSY synchrotrons. Based on previous reports we consider the worst-case scenario of spatial resolution of ~40nm (7). The reconstructed images had cubic voxel dimensions of 20 nm or less. The dimensions of an average size hemozoin crystal in the digestive vacuole, in accordance with hemozoin theoretical growth form (Fig. S1 and ref. (6)) and our observations, is roughly $130\times130\times450=7.6\cdot10^6$ nm^3 (2, 8). There are on average 50 hemozoin crystals in the digestive vacuole of a 30-32 hour old *Plasmodium* parasite (2). The total volume of 50 crystals of this size produces $380\cdot10^6$ nm^3 (=0.38 μm^3) which is expected for a 30-32 hours old *Plasmodium* parasite, where the average hemozoin content measured from soft X-ray

tomography in 46 parasites 30-33 hours old was found to be 0.38±0.02 μm^3 (9). The surface area of such an average crystal is A_{avg}=2·130^2+4·130 ·450=2.68·10^5 nm^2.

Let us consider two extreme cases in which, due to the finite spatial resolution, all dimensions of each crystal are either overestimated or underestimated by 40 nm. For a complete overestimate, the resulting surface area of the average-sized crystal will be A_{+40}=2·170^2+4·170·490=3.91·10^5 nm^2, which is A_{+40} /A_{avg}=1.46 times larger than the given average area. Assuming 95% all overestimates lie within this limit, the standard deviation of the overestimates will be (1.46-1)/2=0.23

For a complete underestimate, the resulting surface area will be A_{-40}=2·90^2+4·90·410=1.64 ·10^5 nm^2, which is A_{-40} /A_{avg}=0.61 of the original area. Here, the standard deviation will be (1-0.6)/2=0.2

The over and the underestimate of the surface area due to the limited resolution completely dominates the 2% error arising from the missing wedge artifact.

The combined standard deviation for the over- and the underestimate is given by $\sqrt{0.23^2 + 0.2^2}$=0.3 or 30 %

3) Precision of Br signal measurement

Br X-ray fluorescence signal collected from the sample is affected by Poisson noise which is at the level of $\sqrt{I_{\mathrm{Br}}}$ The average Br signal level is approximately 40, as given in the main text. Hence the noise level is approximately $\sqrt{40}$=6.3, corresponding to 16% of the average level of Br signal.

4) Overall error in the surface coverage

Since the coverage is inversely proportional to the surface area, the extreme cases translate to 1/1.46=0.68 and 1/0.61=1.64. Again, assuming 95% of measurements lie in these error limits the combined standard deviation is

$\sqrt{((1-0.68)/2)^2 + ((1-1.64)/2)^2} = 0.36$ or 36%

Combined with 16% error in Br signal measurement, the overall error of the surface coverage estimate is given by $\sqrt{0.16^2 + 0.36^2} = 0.39$ or 39%

§4.2 *Number of crystalline unit cells on the surface and in the bulk of isolated hemozoin crystals*

The spatial resolution of X-ray fluorescence maps was estimated at 88 nm full width half maximum (FWHM) as reflected by intensity profiles along and across the needle axis of the hemozoin crystal shown in Fig. 2D. A detailed analysis of the resolution is given in the SI Appendix §6. This resolution is comparable to the transverse dimension of the crystals. Therefore, the determination of the crystal volume and surface area was more elaborate as we present below.

V_{bulk} is the crystal volume. We now derive it from the number of Fe atoms in a crystal measured by X-ray fluorescence. Given that two Fe atoms constitute a unit cell of hemozoin, the crystal volume is equal to $V_{bulk} = (N_{\mathrm{Fe}}/2)v_{\mathrm{uc}}$. Once again, v_{uc} = 1.390 nm^3 is the volume of the unit cell.

The total number of Fe atoms, N_{Fe} is determined from the integrated number of fluorescence counts over pixels belonging to the crystal in the Fe fluorescence map shown in Fig. 2B and detailed in SI Appendix §7.

N_{Fe} in crystal Hz1 determined from four measurements done at different sample orientations is $(2.4\pm0.95)\cdot10^6$. Therefore, the number of unit cells in the whole crystal $N_{bulk} = 2.4\cdot10^6/2 = 1.2\cdot10^6$.

The volume of the isolated crystal Hz1 is

$V_{bulk} = (N_{\mathrm{Fe}}/2)v_{\mathrm{uc}} = (2.4\cdot10^6/2)\cdot1.390 = (1.7\pm0.7)\cdot10^6$ nm^3.

The surface area of the needle-like crystal $S_{\mathrm{Hz}} = L\cdot p_{xs}$, where L=909 nm is the crystal length and p_{xs} its transverse cross-sectional perimeter. The latter may be derived for a given theoretical morphology of a crystal. Assuming a square-like cross-section (10) delineated by the {100} and {010} faces consistent with Fig. S1, the perimeter $p_{xs} = 4\sqrt{V_{bulk}/L}$, where V_{bulk}/L is the corresponding cross-sectional area. The surface area of the isolated crystal Hz1 is therefore given by

$$S_{Hz} = L\cdot p_{xs} = L\cdot4\sqrt{V_{bulk}/L} = 4\sqrt{V_{bulk}\cdot L} = (1.6\pm0.5)\cdot10^5\ \mathrm{nm}^2,$$

which would not change significantly in case of a more rectangular cross-section, as described in the SI Appendix §8.

S_{Hz}, when divided by the weighted average area of surface unit cells, a_{uc}=1.13 nm^2, yields $N_{surf} = (1.5\pm0.5)\cdot10^5$ surface unit cells.

Possible uncertainties in estimates of the volume and the surface area in isolated hemozoin crystals.

The surface area of an isolated crystal is given by $S_{Hz} = 4\sqrt{V_{bulk}\cdot L}$.

Crystal volume is measured from the number of Fe atoms obtained by X-ray fluorescence. For Fe fluorescence intensity of 700, given in the main text, the Poisson noise contributes $\sqrt{700}/700$=0.04 or 4% to the uncertainty in volume definition. This uncertainty translates into ~2% in surface estimate.

On average, the length of an isolated crystal is 800nm (790±180nm as calculated from Table S2). The spatial resolution is 88 nm, resulting in the uncertainty of 88/800=0.11 or 11% in the measurement of the crystal length. This uncertainty translates into ~5% error in the estimate of hemozoin crystal surface area.

§5. *Bromoquine coverage on eight isolated hemozoin crytals*

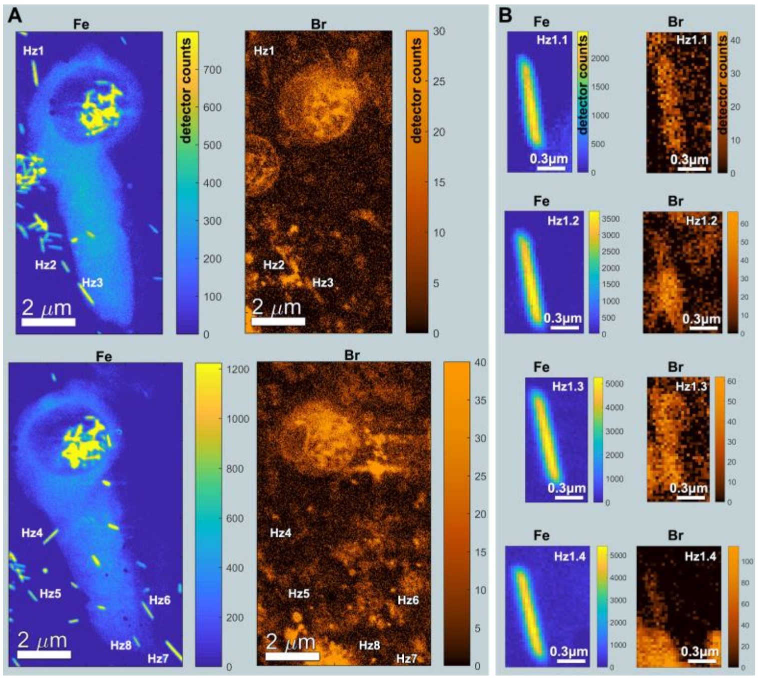

Fig. S3. (A) Eight isolated hemozoin crystals labelled from Hz1 to Hz8 from which BrQ surface coverage and X-ray iron fluorescence signal intensity profiles were measured. The crystals are outside the infected red blood cell, which is not analyzed here. (B) Iron (Fe) and bromine (Br) maps of Hz1 in four different orientations relative to the X-ray beam.

Table S1. Number of iron (#Fe) and bromine (#Br) atoms in hemozoin crystals Hz 1 – Hz 8. *V* - hemozoin volume, Hz length - hemozoin crystal length, Area - surface area of Hz crystal. #UC denotes number of unit cells. #UC labelled "surf" refers to the unit cells on the crystal surface. #UC labelled "BrQ" refers to bromoquine-capped surface unit cells. Covrg is Hz surface coverage by bromoquine as described in the main text.

Hz	#Fe $\times 10^6$	#Br $\times 10^3$	V $\times 10^6$, nm^3	Hz length, nm	Area, $\times 10^3$, nm^2	#UC surf $\times 10^3$	#UC BrQ $\times 10^3$	Covrg, %	<Covrg>, %	± σ, %
1	1.40	2.8	0.95	909	120.0	110.0	5.60	5		
2	0.41	3.4	0.28	480	48.0	45.0	6.80	15		
3	1.30	3.7	0.90	910	120.0	110.0	7.40	7		
4	1.60	2.7	1.10	786	120.0	110.0	5.40	5		
5	0.92	1.4	0.64	545	77.0	72.0	2.80	4	7	±3.5
6	1.30	2.8	0.87	767	110.0	99.0	5.60	6		
7	3.80	9.9	2.60	989	210.0	190.0	19.80	10		
8	0.51	1.1	0.35	454	52.0	49.0	2.20	4		
1.1	1.50	3.5	1.00	941	130.0	120.0	7.00	6		
1.2	2.30	5.1	1.60	938	160.0	150.0	10.20	7		
1.3	3.60	7.1	2.50	928	200.0	180.0	14.20	8		
1.4	3.80	4.8	2.60	933	200.0	190.0	9.60	5		

§6. *Determination of spatial resolution in X-ray fluorescence maps*

Had the spatial resolution in the Fe fluorescence map been much smaller than the crystal dimensions, the projected area of hemozoin a crystal could have been read off directly from iron (Fe) X-ray fluorescence map (see Fig. S3). Since we obtained maps of the crystal Hz1 at four different angles, the total surface area could also have been estimated. However, the spatial resolution was comparable to the transverse dimension of the crystal and therefore the determination of the surface area is somewhat more elaborate as we shall now discuss.

Since crystal Hz1 has sharp edges, the intensity profile of its Fe map (shown in Fig. S3 and Fig. 2B) along the needle axis of Hz1 has approximately trapezoidal shape when convoluted with the resolution. The difference between the long and the short bases of the trapezoid yields the spatial resolution width. This is shown in Fig. S4 and Fig. 2D where the difference in the base edge positions, marked x_1 through x_4 on the horizontal axis, yields a resolution of about 90 nm full width half maximum (FWHM) and a total length of L=909 nm. The intensity profiles for all eight hemozoin crystals are shown in Fig. S4. The trapezoidal coordinates are given in Table S2.

The ideal, theoretical resolution width at the focal position is only about 15 nm (see SI Appendix ref.(12)), but imperfections of the X-ray optics implied a considerably larger width which was measured by knife-edge scans at the focal point to be around 35 nm. However, the Hz1 crystal was not located at the focal point and consequently the FWHM resolution is broadened beyond the focal width, apparently to a value of about 90 nm. The observed horizontal width of 130 nm from the green curve in Fig. 2D could thus well be completely dominated by the resolution, and one cannot estimate the intrinsic width of the crystal with any reasonable degree of accuracy.

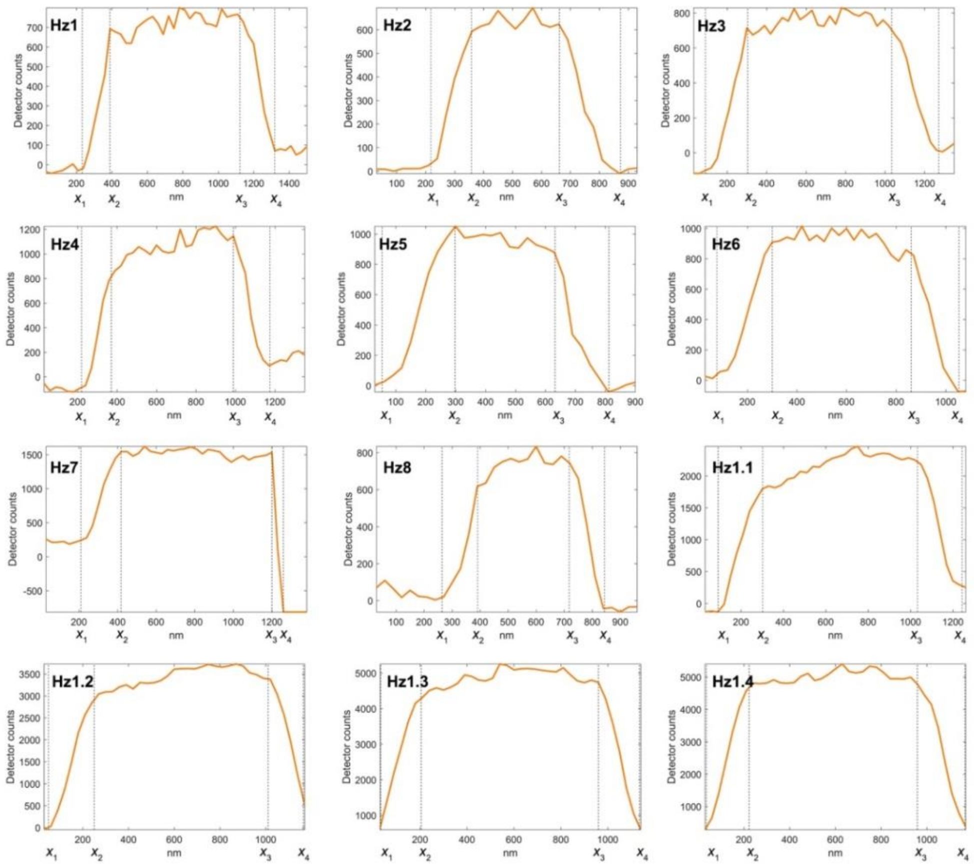

Fig. S4 Profiles of detector counts measured along the needle axis of hemozoin (Hz) crystals. Scans 1 - 8 are acquired with 50ms dwell time with two orientations of the sample grid, 0 and 30 degrees shown in Fig.1 and Fig. S1. Measurements 1.1 through 1.4 are measured from Hz crystal "1" rotated by 0, 15, 30, 45 degrees along its needle axis and with 200 ms dwell time.

Table S2 Trapezoidal coordinates from scans along the needle direction of the Hz crystals 1-8 as plotted in Fig. S3. Δx_{12} and Δx_{34} are the differences between points 1 and 2 and between points 3 and 4 on the detector count profiles shown in Fig. S3. $<\Delta x>$ is the average of all Δx_{12} and Δx_{34}; it represents the full baseline width of the resolution, which is twice the full width at half-maximum (FWHM) resolution.

Hz	x_1, nm	x_2, nm	x_3, nm	x_4 nm	Δx_{12}	Δx_{34}	$<\Delta x>$	$\pm\sigma$
1	233	389	1122	1318	156	196		
2	218	358	662	873	140	211		
3	247	422	1112	1377	175	265		
4	220	371	989	1174	151	185		
5	55	298	632	812	243	180		
6	78	301	861	1053	223	192		
7	211	418	1200		207		187	±34
8	264	391	718	844	127	126		
1.1	92	302	1033	1243	210	210		
1.2	50	250	1011	1165	200	154		
1.3	33	205	960	1135	172	175		
1.4	36	223	959	1167	187	208		

§7. *Conversion of X-ray fluorescence counts into number of iron atoms*
The total number of iron (Fe atoms), N_{Fe} is determined from the integrated number of fluorescence counts CTS_{Fe} over pixels belonging to the hemozoin crystal shown in Fig. 2B. Using the fundamental equation for fluorescent intensity (11) we derive N_{Fe} as follows:

$$N_{Fe} = \frac{\sum_{pixels} \mathrm{CTS}_{Fe}}{I_0(\sigma_{a,Fe}/A_{pixel})\eta_{Fe}(\Delta\Omega/4\pi)}$$

Here I_0 is the incident photon number during exposure of all pixels. The pixel area is denoted by A_{pixel}, the absorption cross-section of an atom by $\sigma_{a,Fe}$, the fluorescence yield by η_{Fe} and the solid angle subtended by the fluorescence detector by $\Delta\Omega$.

§8. *Surface area of a needle-like crystal with a rectangular cross-section*
The transverse cross-sectional area A of a needle-like crystal is determined by the volume V and the length L by $A=V / L$. Assuming a rectangular cross-section of the needle-like crystal with f being the ratio between the long and short edges of the rectangle, the surface area becomes $S_{Hz} = 2\left(f^{1/2} + f^{-1/2}\right)\sqrt{V \cdot L}$ where the geometrical factor $\left(f^{1/2} + f^{-1/2}\right)$ would be 2 for a quadratic cross section having the edge ratio f=1, and 25% more for a rather long rectangle with f=4.

§9. *Analysis of bromine overlap with hemozoin clusters in BrQ-treated and BrQ-free parasites*
We have examined six infected cells treated with BrQ and eight infected cells without BrQ. One BrQ-treated cell and one BrQ-free cell are shown in Fig. S5, the rest are shown in Fig. S6. As explained in detail in Fig. S5 caption, from the Fe map we define a mask called “within Hz” which is composed of a set of pixels with considerable Fe signal. Just outside the periphery of this mask we define another mask labeled “near Hz”. We then calculate the ratio of the average Br signal within each of these two masks. In the ideal case with no statistical fluctuations of intensities, if there is no overlap between Br and Hz, the ratio of intensities should R_I =1. If there is an overlap, the ratio is R_I >1. For eight BrQ-free cells, R_I varied between 0.8 and 1.2, shown with the olive dots in Fig. S5B.

For clarity, we have in the main text defined a term “overlap parameter” $O_{Br,Hz}$ which is equal to R_I-1. This way, when there is no overlap between Fe and Br the overlap parameter $O_{Br,Hz}$=0. If there is an overlap $O_{Br,Hz}$>0. The average overlap parameter $O_{Br,Hz}$ in the BrQ treated cells is 0.57±0.40. In BrQ-free samples it is 0.09±0.12.

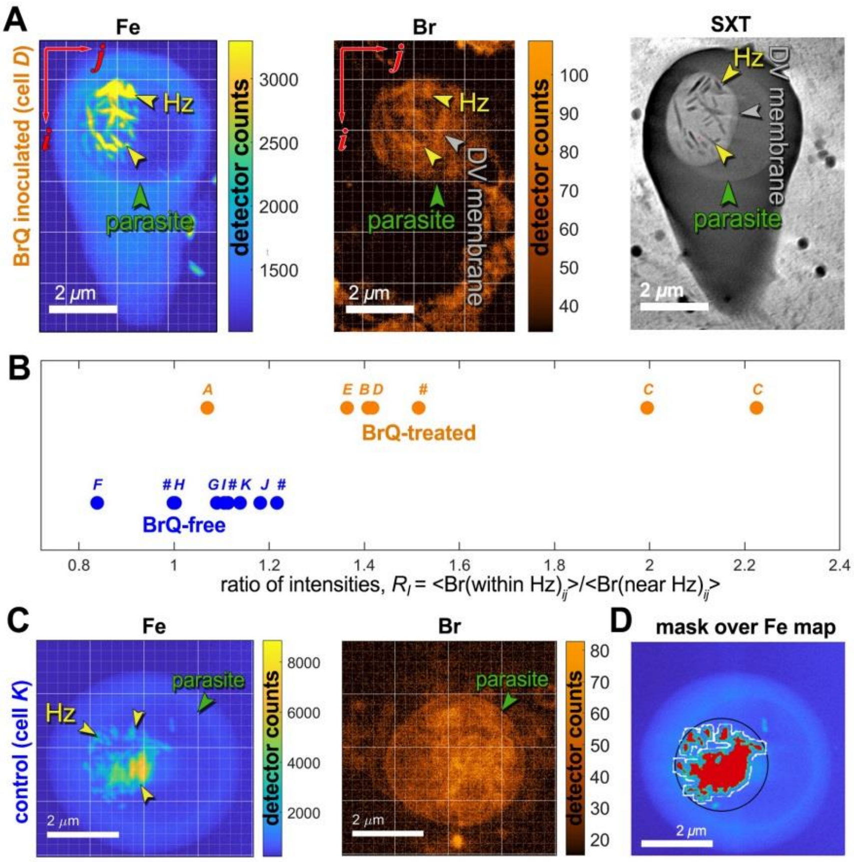

Fig. S5 Overlap between iron (Fe) and bromine (Br) distribution in bromoquine (BrQ) treated and BrQ-free samples

(*A*) Fe and Br fluorescence maps of a BrQ-treated infected red blood cell (iRBC) next to a tomographic section through the middle of its parasite imaged by soft X-ray cryo-tomography (SXT). DV denotes digestive vacuole, Hz denotes hemozoin crystals, *i*,*j* are pixel coordinates.

(*B*) Overlap between Hz position and Br fluorescence signal in BrQ-treated and BrQ-free samples. Each dot represents a measurement within an individual parasite carrying a name of its host iRBC. iRBCs shown in Fig. S6 are named *A* - *K*, others are labelled with a hashtag. Cells *C* and *A* are shown in Figs. 1 and 2, respectively. The correlation is expressed in terms of the average Br count in pixels "within" Hz crystals divided by the average Br count in pixels near Hz. The ratio is larger than one if there is an elevated Br signal at the *i*,*j* pixel coordinates within Hz compared to that just outside Hz.

(*C*) Fe and Br signals in a BrQ-free sample.

(*D*) An overlay of the Fe map from panel *C* and the masks labelled "within Hz" colored red, and "near Hz", the white line of pixels. An average Fe count within the circle containing Hz is calculated. The red colored pixels are those where the Fe fluorescence intensity is higher than this average, they form the mask labelled "within Hz". The white line is a line of pixels at a fixed small distance from the red mask – these pixels are labelled "near Hz". For cell *K* in panel *C* the average of the Br intensities within the red mask relative to the average within the white mask turned out to be 1.17 as indicated by the blue dot marked "*K*" in panel *B*. The same procedure was carried out for a cell treated with BrQ with the result of 1.42 as indicated by the orange dot marked "*D*" in panel *B*. The larger value for cell *D* than for *K* indicates tendency for BrQ to accumulate on Hz crystals.

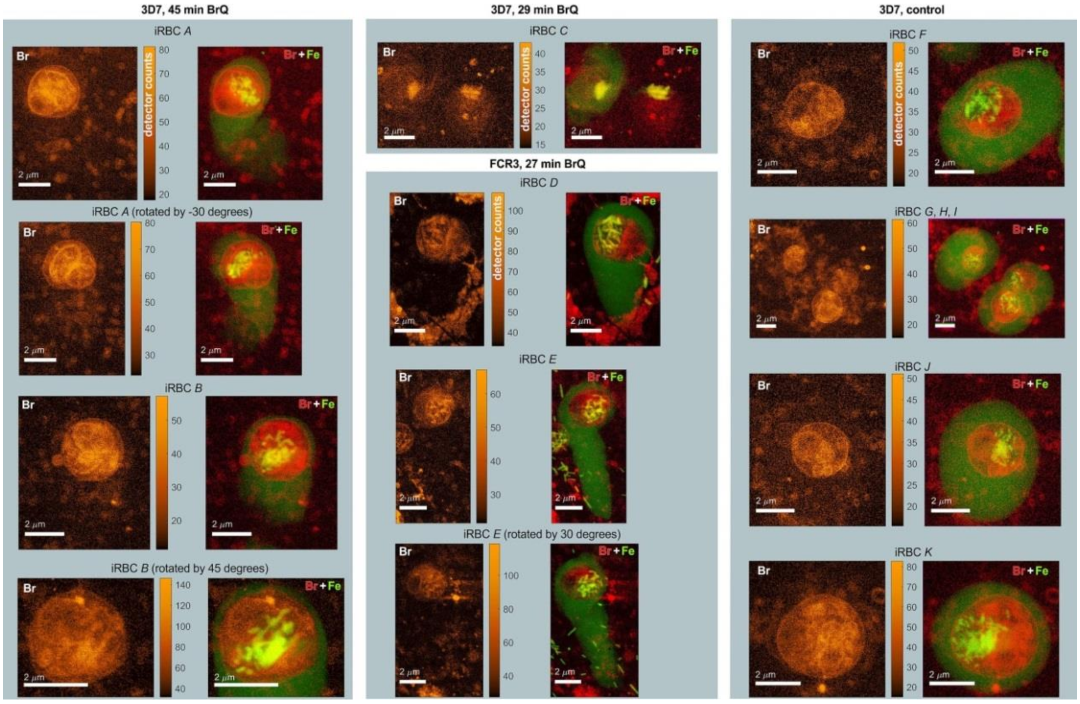

Fig. S6 *Left four columns*: Distribution of bromine (Br) and overlay of Br and iron (Fe) maps in bromoquine (BrQ)-treated samples of red blood cells infected with chloroquine susceptible (3D7) and resistant (FCR3) strains of *Plasmodium falciparum* parasites. The samples are labeled A-E. The time in minutes elapsed between BrQ introduction and sample vitrification is indicated. Different orientations of the samples relative to the X-ray beam are also indicated *Right two columns*: Br and overlay Br/Fe maps of BrQ-free samples. These samples are labeled F-G. Iron and sulfur maps of the same cells are shown in Fig. S7

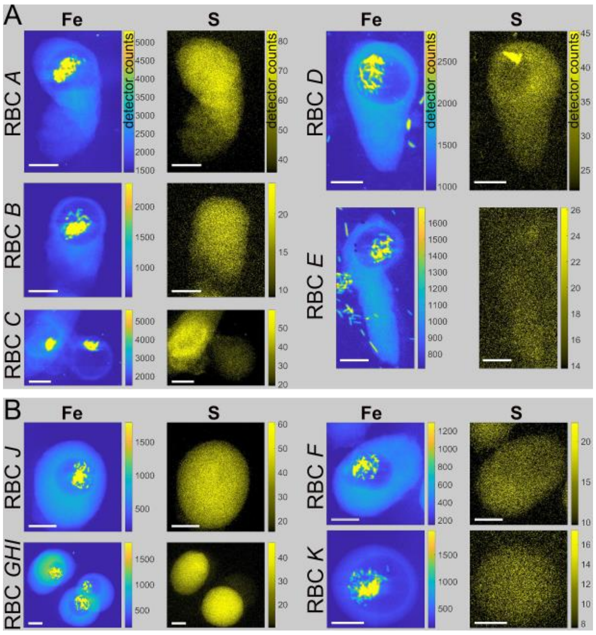

Fig. S7 (A) Fluorescence intensity maps of Fe and S for BrQ-treated cells *A–E*. Elevated iron signal over RBC cytosol area correlates with elevated S signal over the same area since a hemoglobin molecule contains 12 S atoms and 4 Fe atoms (11). As such, the ratio of S:Fe in RBC cytosol should be ~3, which is indeed observed (3±0.4) for cells A-D. However, due to high absorption of X-ray fluorescence signal from S in the ice formed upon the vitrification of the RBC the measured ratio is not precise, and for cell *E* is dominated by noise. Note, the fluorescent intensity per S atom is lower than that per Fe atom by a factor of ~39.5, such that $\frac{N_{\mathrm{S}}}{N_{\mathrm{Fe}}} \approx 39.5 \frac{I_{\mathrm{S}}}{I_{\mathrm{Fe}}} e^{L/25}$, where N is the number of atoms, I – measured fluorescent intensity, the exponential term is compensation for absorption of *S* signal in ice with *L* being the fluorescent X-ray beam path length in the ice.

(B) Fluorescence intensity maps of Fe and S for BrQ-free cells *F–K*. Ice thickness was not measured for these cells.

§10 *Comparison of Br distribution in chloroquine-resistant and sensitive strains*
BrQ was introduced both to chloroquine sensitive 3D7 and chloroquine resistant FCR3 strains of the parasite at the concentration of 40 nM, except for one case (iRBC *C*, 3D7 strain) where BrQ concentration was chosen to be 150 nM. To our surprise, a close inspection of these maps reveals similar Br distribution in both strains. The reason is not obvious to us. We try to rationalize this observation by noting that BrQ was introduced into the FCR3 culture at the concentration of 40 nM which might be too close to the measured 62 nM IC_{50} value of BrQ for FCR3.

The IC_{50} values of BrQ and chloroquine measured for FCR3 and 3D7 strains are given in SI Appendix §15, Table S3. A more systematic study with several different BrQ concentrations and incubation times shall be conducted to address this observation.

§11. *Estimate of hemozoin crystal coverage by free bromoquine in the digestive vacuole of an early trophozoite*
The average concentration of free bromoquine (BrQ) in the digestive vacuole is [BrQ]=150μM. Let us estimate hemozoin surface coverage in a young trophozoite, two hours after hemozoin crystals begin to form. We assume the volume of digestive vacuole, V_{DV}, of such a trophozoite is approximately two femtoliters, see ref. (2). If half of the digestive vacuole is occupied by BrQ, then there will be $N_{BrQ} = [\mathrm{BrQ}] \cdot N_A \cdot 0.5 \cdot V_{DV} = 150 \cdot 10^{-6} \cdot 6.022 \cdot 10^{23} \cdot 0.5 \cdot 2 \cdot 10^{-15} = 90000$ free BrQ molecules, where N_A is Avogadro's number.

Let us assume that after two hours or 7200 sec 15 hemozoin crystals are formed. At the total rate of hemozoin formation of 3500 unit cells per second (13) the average volume of each crystal will be $V_{Hz} = 7200 \cdot 3500 \cdot V_{UC}/15 = 2{,}335{,}000$ nm^3, where $V_{UC} = 1.39$ nm^3 is the volume of the hemozoin crystal unit cell. Assuming a needle-like shape with a square cross-section and a ratio of crystal length to width of 4:1, similar to that of the theoretical growth form of hemozoin, at the given volume V_{Hz}, the crystal width will be $D_{Hz} = \left(\frac{V_{Hz}}{4}\right)^{1/3} \approx 83.6$ nm. The crystal area would therefore be $A_{Hz} = 2 \cdot \left({D_{Hz}}^2\right) + 4 \cdot \left(D \cdot (4 \cdot D)\right) \approx 1.26 \cdot 10^5$ nm^2. The average area of the surface unit cell of hemozoin $\bar{A}_{\mathrm{UC}} = 1.3$ nm^2. The number of unit cells on hemozoin crystal surface is $A_{Hz}/\bar{A}_{\mathrm{UC}} = 1.26 \cdot 10^5$. Given that a BrQ molecule spans two neighboring surface unit cells when docked onto a hemozoin crystal, the number of docking sites on a single hemozoin crystal is $1.26 \cdot 10^5/2 \approx 55700$. The total number of docking sites on all 15 crystals is therefore 835000. The coverage can therefore be estimated as 90000/835000 ≈ 0.108, or ~11%.

§12. *Synthesis of Bromoquine.*
The synthesis of bromoquine (BrQ), was performed making use of a modified version of the reported procedure (14). In a 50 mL round bottom flask, 7-bromo-4-chloroquinoline (10 mmol, 2.42g) and 2-amino-5-diethylaminopentane (17 mmol, 3.28 mL) were added and heated at 85 ^{o}C with stirring. After 1 h, the reaction temperature was elevated to 140 ^{o}C for 20 h and the reaction was monitored by TLC

(thin layer chromatography) until completion. The reaction mixture was then cooled to 90 °C, and excess amounts of the diamine was evaporated under reduced pressure. After cooling to room temperature, aq. NaOH (1 N, 20 mL) was added and then organic layer was extracted with CH_2Cl_2 (3 x 40 mL). The collected organic phase was washed with brine, and then dried over anhydrous Na_2SO_4 and concentrated under reduced pressure to afford crude product (brown oil). The product (BrQ) was obtained after column chromatography (MeOH/CH_2Cl_2 = 1:3, R_f = 0.25) as an off-white powder (1.27 g, 35%). ^{1}H NMR (500 MHz, $CDCl_3$) δ = 8.50 (d, J = 5.7 Hz, 1H), 8.11 (q, J = 2.3 Hz, 1H), 7.62 (t, J = 7.6 Hz, 1H), 7.46 (dt, J = 9.6, 3.4 Hz, 1H), 6.42 (t, J = 6.0 Hz, 1H), 5.31 (d, J = 6.7 Hz, 1H), 3.70 (q, J = 6.4 Hz, 1H), 2.54 (p, J = 7.0 Hz, 4H), 2.46 (q, J = 6.8 Hz, 2H), 1.75 (m, J = 6.7 Hz, 1H), 1.70 – 1.48 (m, 3H), 1.30 (t, J = 6.4 Hz, 3H), 1.01 (q, J = 6.9 Hz, 6H). ^{13}C NMR (126 MHz, $CDCl_3$) δ = 152.09, 149.79, 149.25, 132.28, 127.65, 123.12, 121.44, 117.80, 99.49, 52.68, 48.44, 46.97, 34.63, 23.88, 20.32, 11.39. HRMS: *m/z* calculated for $C_{18}H_{27}BrN_3$ $[M+H]^+$: 364.13829; found: 364.13813. The NMR spectrum of BrQ is presented in Fig. S8.

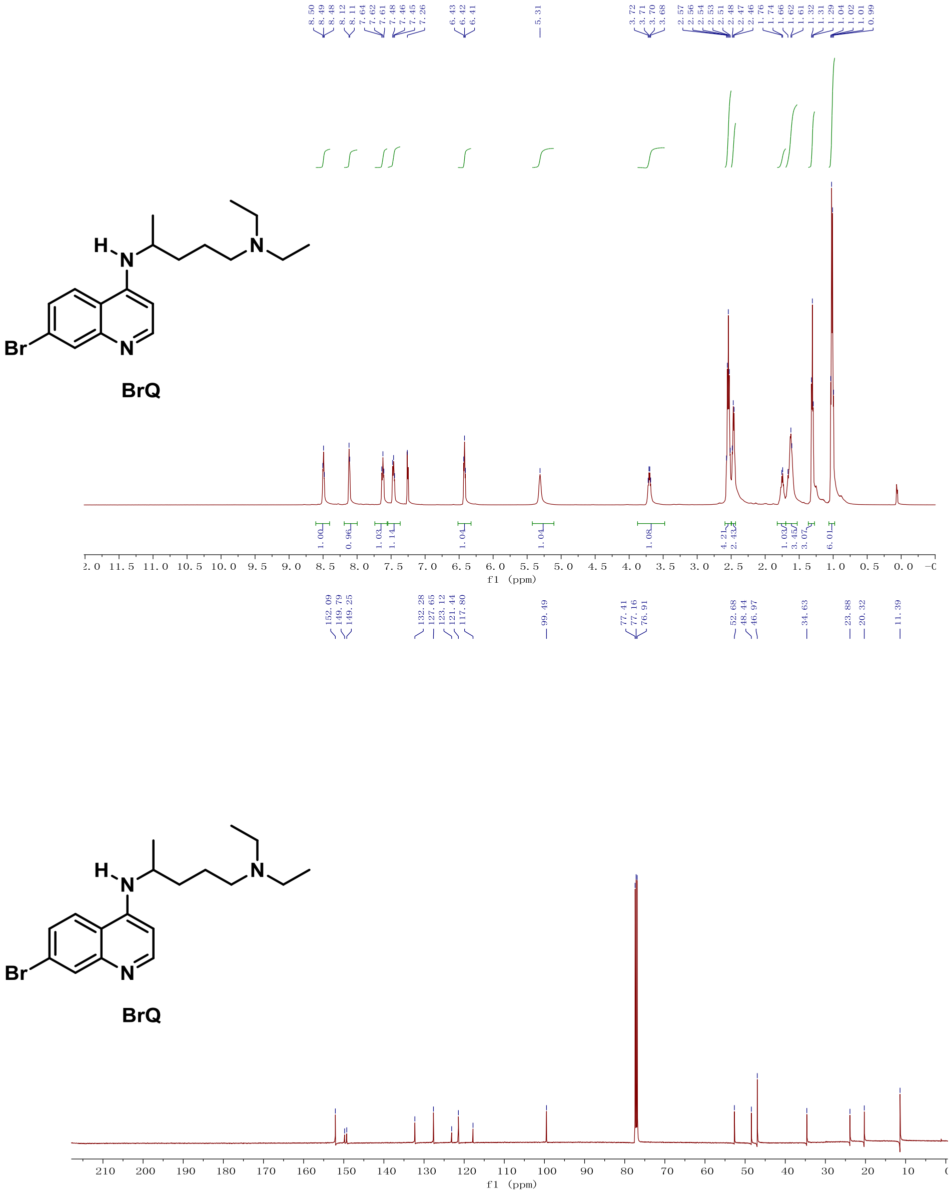

Fig. S8 NMR spectrum of bromoquine

§13. *Malaria parasite culture*

Plasmodium falciparum (3D7 and FCR3 strains) blood stage parasites were cultured *in vitro* in human type 0 Rh+ erythrocytes at 4% hematocrit in HEPES buffered RPMI1640 (Sigma) medium supplemented with 5g/l Albumax II (Gibco), 20mg/l hypoxanthine, 180mg/l L-glutamine and 50mg/l gentamicin. Cultures were incubated at 37°C in an atmosphere of 2% O_2, 5,5% CO_2 and 92,5% N_2, as described. (15)

The genetic identity of 3D7 and FCR3 were controlled by typing of line-specific polymorphisms in the MSP1 and MSP2 loci (16).

§14. *Synchronization of malaria parasites*

Tight synchronization of malaria parasites growth was achieved by purifying erythrocytes infected by malaria parasites older than 20h post invasion from an asynchronous culture by magnet activated cell sorting (MACS) (Milteney Biotech) (17, 18) using a 20G needle to regulate flowrate. Purified 20-48h old parasites were then incubated for 2h with uninfected red blood cells at approximately 20% hematocrit in culture medium at 35°C on a rocking table, to optimize bursting of the schizonts and invasion of the uninfected erythrocytes. After the 2h incubation period remaining late stage parasites were removed from the erythrocyte mixture by another passage through a MACS column using a 22G needle to obtain a lower flow rate optimal for depletion. Microscopy of the erythrocytes depleted for parasites older than 20h revealed that >95% of the remaining parasites were young ring stages that had recently invaded their host cell. These were incubated under standard culture conditions for 30h. The resulting 30-32h parasites were then separated from the uninfected erythrocytes in the culture by repeating the initial MACS purification step.

§15. *Bromoquine and chloroquine IC_{50} values for 3D7 and FCR3 strains*

Parasite cultures were diluted to a starting parasiteamia of 0.5% and a hematocrit of 2% (4μl packed erythrocytes in a total volume of 200μl) with different concentrations of chloroquine and bromoquine in 96 well flat bottom NUNC culture plates. The plates were incubated for 48 hours at standard parasite culturing conditions. Final parasitemia was evaluated by staining cultures with acridine orange and counting infected red blood cells by flow cytometry as previously described by Hein-Kristensen *et al*. (19). Assays were run in triplicate. The measurements are shown in Fig. S9. Error bars shown in graphs are standard deviations of triplicates. The measured IC_{50} values are given in Table S3.

Table S3 IC_{50} values for bromoquine (BrQ) and chloroquine CQ in chloroquine-sensitive (3D7) and chloroquine-resistant (FCR3) strains

	3D7	FCR3
CQ	14 nM	105 nM
BrQ	10 nM	62 nM

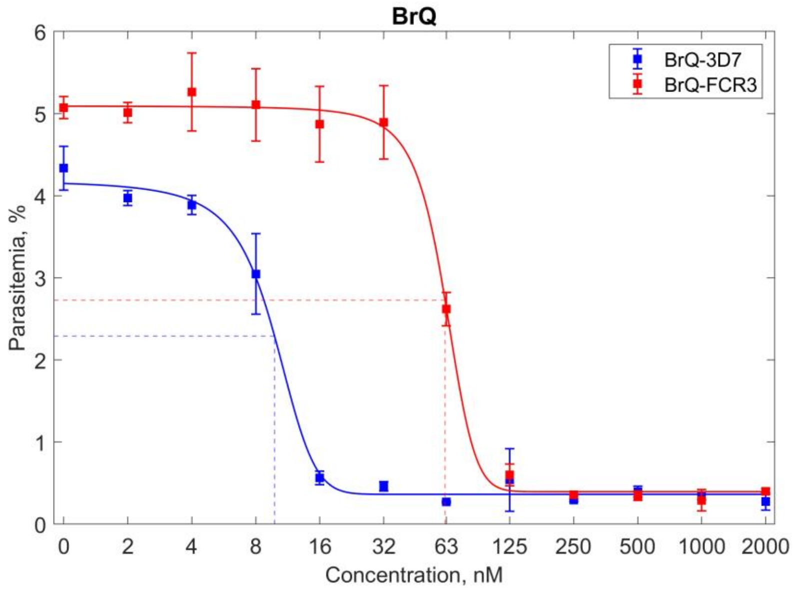

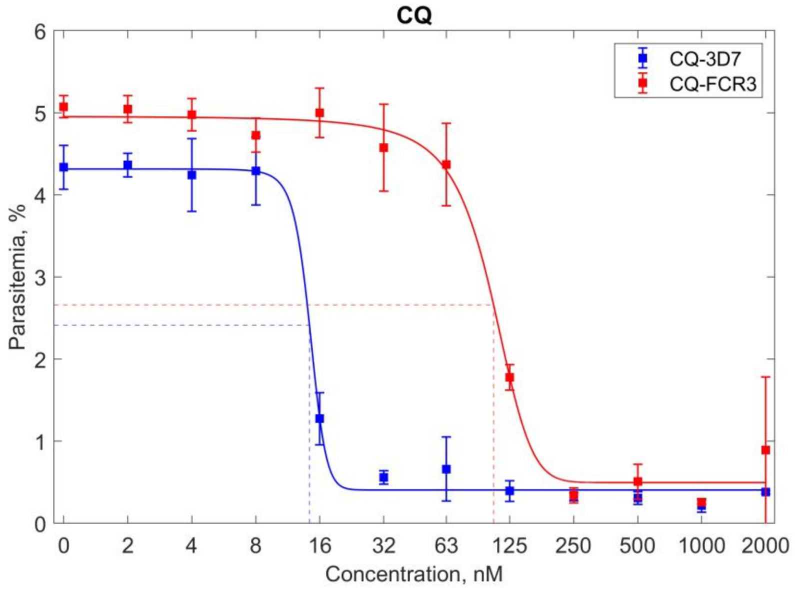

Fig. S9 Inhibition of chloroquine sensitive (3D7) and chloroquine resistant (FCR3) strains by chloroquine (CQ) and bromoquine (BrQ).

§16. *Sample vitrification*

A drop of 1-3 μl of the parasite culture was placed onto a 3mm electron microscopy specimen grid made of aluminum, sandwiched between two aluminum disks and rapidly vitrified by high pressure freezing using Leica HPM100 setup at the Core Facility for Integrated Microscopy, University of Copenhagen.

Drug treatment

Bromoquine drug was added to the parasite culture placed in the PBS buffer at concentrations of 40nM or 150nM. Before and after treatment with the drug the parasite culture was kept at 37°C. The culture samples were then vitrified at 27, 29 and 45 minutes after the treatment with the drug resulting in well-defined duration of the drug action.

§17. *The X-ray imaging instruments*

Cryo X-ray fluorescence mapping was conducted at ID16A beamline at ESRF. The X-ray source at the beamline ID16A at ESRF is a 28 mm period, in-vacuum undulator using the 3rd harmonic at 17.05 keV.

The focusing of the beam was provided by a KB set of aspherical, multilayer W/BC4 coated mirrors(20) with a 1% relative energy bandwidth at a distance of 185 m from the source. In the vertical plane the center of the mirror was 0.1 m from the image point so the geometrical demagnification was 0.1m/185m = 0.54×10^{-3}. The relatively large horizontal electron beam size in ESRF necessitated a virtual beamspot 40 m downstream from the undulator and a resulting geometrical demagnification of 0.045m/145m = 0.3110^{-3}. Imperfections in the KB mirrors implied a FWHM beamsize at the focal point of about 30-40 nm, considerably larger than the ideal theoretical width of about 15 nm, which actually has been obtained experimentally at the same beamline with another KB system designed for 33.6 keV (*cf.* da Silva *et al.* (12)). The resulting intensity at 17.05 keV was around 10^{11} photons/sec. The six X-ray fluorescence detectors subtended altogether a solid angle $\Delta\Omega$ of 0.4. A typical scan in the (i, j) plane comprised 200 x 200 pixels, each 30 x 30 nm, and took less than one hour using 0.1 sec per point.

Soft X-ray cryo-tomography (SXT) measurements of cells *A* and *B* (*cf.* Figs. 4 and S5) were conducted at MISTRAL beamline at ALBA synchrotron light source. SXT of cells *C*, *D* and *E* (Figs. 1, 2, 3 and S6) were conducted at U41-PGM1-XM beamline at BESSY-II synchrotron at Helmholtz Zentrum Berlin.

The MISTRAL beam line at ALBA uses a bending magnet as source (7). The transmission X-ray microscope (TXM, Zeiss, formerly Xradia) was set at a photon energy of 520 eV. A single-reflection elliptical glass capillary condenser focuses monochromatic light on to the sample, which is an EM grid at cryo-temperature. The transmitted signal is collected by an objective Fresnel zone plate of 25 nm outermost zone width, and a magnified image is delivered to a direct illumination CCD camera. The spatial resolution in 2D is ~26 nm (21) for tomography using a tilt range from -60 to 60 degree.

The U41-PGM1-XM beamline BESSY-II uses an undulator as source, a plane grating monochromator and a single-bounce elliptical capillary as condenser (22, 23). The transmission X-ray microscope was set to a photon energy of 510 eV. A Fresnel zone plate with a 25 nm outermost zone width was used to image cryogenic samples maintained at -170°C within a tilt range of -60 to +60 degrees. The pixel size in the magnified images is 9.8 nm and the 3D resolution is about 36 nm.

§18. *Software*

Data analysis was done by custom-written programs in MATLAB. Soft X-ray cryo-tomography projection series alignment was done in Bsoft (24). The aligned projections were reconstructed with Tomo3D (25) or TomoJ (26). Volume segmentation of reconstructed tomograms was done in Medical Imaging Interaction Toolkit (MITK) (27). Segmented volume was visualized by surface rendering in 3D Slicer (28). PyMCA software package was used to calculate the fluorescence spectrum for a fitted amount of irradiated fluorescent atoms (29).

References

1. Straasø T, *et al.* (2014) The Malaria Pigment Hemozoin Comprises at Most Four Different Isomer Units in Two Crystalline Models: Chiral as Based on a Biochemical Hypothesis or Centrosymmetric Made of Enantiomorphous Sectors. *Crystal Growth & Design* 14(4):1543-1554.
2. Kapishnikov S, *et al.* (2012) Oriented nucleation of hemozoin at the digestive vacuole membrane in *Plasmodium falciparum*. *Proc. Natl. Acad. Sci. U. S. A.* 109:11188 – 11193.
3. Als-Nielsen J & McMorrow D (2011) Figure 7.1. *Elements of Modern X‐ray Physics*, (Wiley), Second Edition Ed.
4. Henke BL, Gullikson EM, & Davis JC (X-ray interactions: photoabsorption, scattering, transmission, and reflection at E = 50–30,000 eV, Z = 1–92. Atomic Data and Nuclear Data Tables, pp 181-342.
5. Bambynek W, *et al.* (1972) X-Ray Fluorescence Yields, Auger, and Coster-Kronig Transition Probabilities. *Reviews of Modern Physics* 44(4):716-813.
6. Buller R, Peterson ML, Almarsson O, & Leiserowitz L (2002) Quinoline Binding Site on Malaria Pigment Crystal: A Rational Pathway for Antimalarial Drug Design. *Cryst Growth Des* 2:553-562.
7. Sorrentino A, *et al.* (2015) MISTRAL: a transmission soft X-ray microscopy beamline for cryo nano-tomography of biological samples and magnetic domains imaging. *Journal of Synchrotron Radiation* 22(4):1112-1117.
8. Kapishnikov S, *et al.* (2012) Aligned hemozoin crystals on a curved surface in malarial red blood cells revealed by nanoprobe X-ray Fe-fluorescence and diffraction. *Proc. Natl. Acad. Sci. U. S. A.* 109:11184 – 11187.
9. Magkos S (2018) Decoding structure and composition using multi-scale X-ray and neutron imaging techniques. M.Sc (Faculty of Science, University of Copenhagen, Copenhagen, Denmark).
10. Noland GS, Briones N, & Sullivan D (2003) The shape and size of hemozoin crystals distinguishes diverse Plasmodium species. *J. Mol. Biochem. Parasitol.* 130:91-99.
11. Kapishnikov S, *et al.* (2017) Biochemistry of malaria parasite infected red blood cells by X-ray microscopy. *Scientific Reports* 7(1):802.
12. Cesar da Silva J, *et al.* (2017) Efficient concentration of high-energy x-rays for diffraction-limited imaging resolution. *Optica* 4(5):492-495.
13. Kapishnikov S, *et al.* (2017) Unraveling heme detoxification in the malaria parasite by *in situ* correlative X-ray fluorescence microscopy and soft X-ray tomography. *Scientific Reports* 7(1):7610.
14. De D, Krogstad FM, Byers LD, & Krogstad DJ (1998) Structure−Activity Relationships for Antiplasmodial Activity among 7-Substituted 4-Aminoquinolines. *Journal of Medicinal Chemistry* 41(25):4918-4926.
15. Cranmer SL, Magowan C, Liang J, Coppel RL, & Cooke BM (1997) An alternative to serum for cultivation of Plasmodium falciparum in vitro. *Transactions of the Royal Society of Tropical Medicine and Hygiene* 91(3):363-365.
16. Wang CW, *et al.* (2012) Evidence for in vitro and in vivo expression of the conserved VAR3 (type 3) plasmodium falciparum erythrocyte membrane protein 1. *Malaria Journal* 11(1):129.
17. Ribaut C, *et al.* (2008) Concentration and purification by magnetic separation of the erythrocytic stages of all human Plasmodium species. *Malaria Journal* 7(1):45.

18. Paul F, Roath S, Melville D, Warhurst DC, & Osisanya JOS (1981) Separation of malaria-infected erythrocytes from whole blood: use of a selective high-gradient magnetic separation technique. *Lancet* 2:70-71.
19. Hein-Kristensen L, Wiese L, Kurtzhals JAL, & Staalsoe T (2009) In-depth validation of acridine orange staining for flow cytometric parasite and reticulocyte enumeration in an experimental model using Plasmodium berghei. *Experimental Parasitology* 123(2):152-157.
20. Morawe C*, et al.* (2015) *Graded multilayers for figured Kirkpatrick-Baez mirrors on the new ESRF end station ID16A* p 958803.
21. Oton J*, et al.* (2016) Characterization of transfer function, resolution and depth of field of a soft X-ray microscope applied to tomography enhancement by Wiener deconvolution. *Biomed Opt Express* 7(12):5092-5103.
22. Guttmann P, Werner S, Rehbein S, Habel C, & Schneider G (2018) First Results from the X-Ray Microscopy Beamline U41-PGM1-XM at BESSY II. *Microscopy and Microanalysis* 24(S2):202-203.
23. Guttmann P*, et al.* (2018) The New HZB X-Ray Microscopy Beamline U41-PGM1-XM at BESSY II. *Microscopy and Microanalysis* 24(S2):204-205.
24. Heymann J, Cardone G, Winkler D, & Steven A (2008) Computational resources for cryo-electron tomography in Bsoft. *J. Struct. Biol.* 161:232–242.
25. Agulleiro JI & Fernandez JJ (2010) Fast tomographic reconstruction on multicore computers. *Bioinformatics* 27(4):582-583.
26. Messaoudi C, Boudier T, Sorzano COS, & Marco S (2007) TomoJ: tomography software for three-dimensional reconstruction in transmission electron microscopy. *BMC Bioinformatics* 8:288-297.
27. Wolf I*, et al.* (2004) The medical imaging interaction toolkit (MITK): a toolkit facilitating the creation of interactive software by extending VTK and ITK. *Medical Imaging 2004*, (SPIE), p 12.
28. Fedorov A*, et al.* (2012) 3D Slicer as an image computing platform for the Quantitative Imaging Network. *Magnetic resonance imaging* 30(9):1323-1341.
29. Solé VA, Papillon E, Cotte M, Walter P, & Susini J (2007) A multiplatform code for the analysis of energy-dispersive X-ray fluorescence spectra. *Spectrochimica Acta B* 62:63-68.
